# Regulation of HNRNP family by post-translational modifications in cancer

**DOI:** 10.1038/s41420-024-02198-7

**Published:** 2024-10-04

**Authors:** Bohao Li, Mingxin Wen, Fei Gao, Yunshan Wang, Guangwei Wei, Yangmiao Duan

**Affiliations:** 1 Department of Cell Biology and Key Laboratory of Experimental Teratology, Ministry of Education, School of Basic Medical Sciences, Cheeloo College of Medicine, Shandong University, Jinan, Shandong China; 2 Department of Anatomy, School of Basic Medical Sciences, Cheeloo College of Medicine, Shandong University, Jinan, Shandong China; 3 Department of Clinical Laboratory, Shandong Provincial Hospital Affiliated to Shandong First Medical University, Jinan, Shandong China

**Keywords:** Post-translational modifications, Post-translational modifications

## Abstract

Heterogeneous nuclear ribonucleoproteins (HNRNPs) represent a large family of RNA-binding proteins consisting of more than 20 members and have attracted great attention with their distinctive roles in cancer progression by regulating RNA splicing, transcription, and translation. Nevertheless, the cancer-specific modulation of HNRNPs has not been fully elucidated. The research of LC-MS/MS technology has documented that HNRNPs were widely and significantly targeted by different post-translational modifications (PTMs), which have emerged as core regulators in shaping protein functions and are involved in multiple physiological processes. Accumulating studies have highlighted that several PTMs are involved in the mechanisms of HNRNPs regulation in cancer and may be suitable therapeutic targets. In this review, we summarize the existing evidence describing how PTMs modulate HNRNPs functions on gene regulation and the involvement of their dysregulation in cancer, which will help shed insights on their clinical impacts as well as possible therapeutic tools targeting PTMs on HNRNPs.

## Facts

- The HNRNP protein family forms a structurally conserved and functionally highly correlated protein subgroup that is involved in many important cellular life activities.
- HNRNPs contribute to pathogenesis and progression of tumors by facilitating cancer cell proliferation, migration, metabolic dysregulation, and other related factors.
- With the development of the LC-MS/MS technology, the HNRNPs proteins were identified to target by widespread and multiple modifications.
- Protein modifications can mediate multiple biological functions of HNRNPs such as structure, subcellular localization, and protein interactions, and then regulate cancer development.

## Open questions

- How many types of PTMs are identified mapped to HNRNP family in cancer?
- What roles do protein modifications play in the functions of HNRNPs and cancer progression?
- Do protein modifications have practical application prospects in the diagnosis and treatment of cancers?

## Introduction

Cancer is a severely detrimental disease to human health and imposes a significant burden on human society [1]. Over the years, we have committed to unravel the mechanisms of cancer progression and seek innovative therapeutic strategies. However, the refractory, recurrent, and augmented survival capacities of tumor cells continue to pose significant challenges in cancer therapy for the future. It is noteworthy that the HNRNP family composed of a series of RNA-binding proteins (RBPs) participates in regulation of crucial biological processes, including RNA splicing, translation, and mRNA localization [2, 3]. Studies have suggested that most HNRNPs negatively regulate splicing function, and they combine with cis-acting elements intronic splicing silencer (ISS) or exonic splicing silencer (ESS) to inhibit the activity of adjacent splicing sites or the ability of splicing bodies to recognize adjacent splicing sites [4]. These RBPs were shown to be created out of about 20 types of HNRNPs, named from A to U, and the expression levels of these HNRNPs are higher in most cancer cells than corresponding normal cells. Additionally, the respective members of the HNRNP family contribute to pathogenesis and progression of tumors by facilitating cancer cell proliferation, migration, metabolic dysregulation, inflammation, and other related factors [4–7]. The intensive studies of the HNRNPs regulation in cancer will help us find the potential therapeutic targets of cancers.

Post-translational modifications (PTMs) covalently bond small molecular groups on the amino acid side chain of proteins with broad function distributions, which contain diverse types such as phosphorylation, ubiquitination, glycosylation, and acylations including succinylation, hydroxyisobutyrylation, nitrosylation, lactylation [8–11]. These PTMs can mediate the protein functions in multiple cellular biological processes and subsequently influence physiological processes [11]. With the development of the high-resolution liquid chromatography coupled with tandem mass spectrometry (LC-MS/MS) [9], the HNRNP family was identified as targets of widespread and multiple modifications, including the phosphorylation in serine and threonine residues, arginine methylation, as well as SUMOylation and glycosylation processes. These modifications can induce alterations in the structure, subcellular localization, and protein interactions, thereby impacting the cancer development [12–14]. This review offers a broad overview of the regulation of HNRNPs by PTMs and the involvement of their dysregulation in cancer.

## HNRNP family

The HNRNP complexes were initially identified as composed of *A1*, *A2/B1*, *C1*, and *C2* in human HeLa cells, which were designated as core HNRNP particles [15]. These HNRNPs have high affinity and specificity for pre-mRNA, forming the fundamental 40S HNRNP-pre-mRNA complexes. In addition to core HNRNP particles, the HNRNP family was identified as composed of multiple RBPs and referred to as minor HNRNP. Minor HNRNP members have relatively low binding ability to pre-mRNA [8]. The protein molecular weights of these HNRNP family members range from 34 to 120 kDa, and names from A1 to U [16]. The HNRNPs bind to ESSs or ISSs of pre-mRNA, thereby stabilizing mRNA and affecting mRNA localization, which facilitates the generation of inflammatory splicing subtypes or increase resistance-related splicing subtypes [17–19].

The majority of HNRNPs have three fundamental structural domains-the RNA recognition motif (RRM), the Arginine-Glycine-Glycine (RGG), and the KH domain (KH). Several HNRNPs also contain quasi-RNA recognition motif (qRRM), nuclear localization signal (NLS), nucleo-plasmic shuttle domain (NS), and Zinc Finger (ZnF) domains. Moreover, a significant proportion of HNRNPs exhibit glycine-rich residues at their C-terminal region. In eukaryotic organisms, RNA Polymerase II mediates the release of mRNA transcripts [20]. Precise splicing on pre-mRNA leads to the generation of mature mRNA through alternative splicing (AS), which contributes to the remarkable diversity within the human proteome [21]. AS is specifically regulated by spliceosomes and other RNA-binding proteins, which act as splicing factors, such as serine and arginine-rich (*SR*) proteins, the HNRNP family proteins, and a diverse array of proteins featuring RRM, KH, and basic leucine zipper (bZIP) structures. It is widely demonstrated that SR and HNRNP proteins exhibit antagonistic roles, and *SR* proteins are splicing activators, while HNRNP proteins are splicing repressors. The splicing process is precisely regulated by the interaction between SRs and HNRNPs with a concentration-dependent manner [4, 22].

## PTMs in cancer

**Fig. 1:**
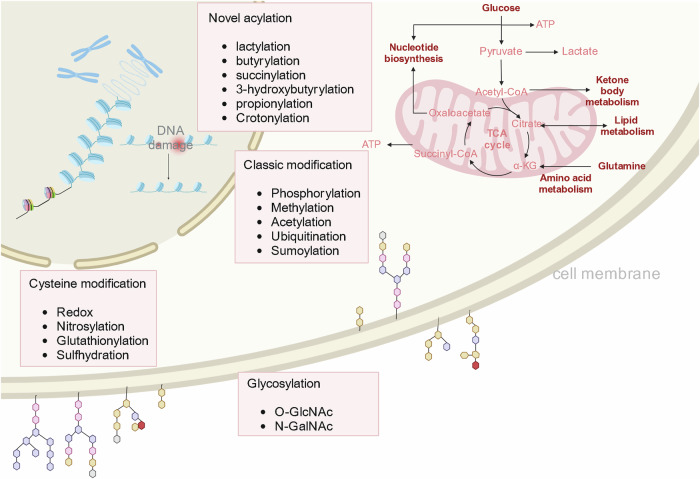
Types of PTMs (created with BioRender.com). (1) classical modifications that are abundant in cells and play key roles in cellular energy metabolism, lipid metabolism, ketone body metabolism, and amino acid metabolism. (2) Modifications present only in cysteine. When cells are subjected to oxidative stress, oxidation modification on cysteine may occur as a response. (3) Membrane proteins glycosylation plays an important role in the protection, stability, and barrier of cell membranes. (4) Novel acylation modifications are involved in epigenetic regulation, which plays a role in DNA damage repair, histone acylation modification, chromatin packaging, and so on.

PTM refers to the covalent attachment of molecular groups to amino acid side chains in proteins, which leads to the alteration or modulation of protein conformational structure as well as their activities and physicochemical properties (Fig.1). Ultimately, these modifications exert a profound impact on crucial cellular processes including cell cycle, transcription, metabolism, immunity, and autophagy. Consequently, they play an indispensable role in cell survival, proliferation, migration, differentiation, and apoptosis [23]. Currently, the techniques for characterizing and analyzing PTMs are relatively mature. The LC-MS/MS technique was first used in the early 2000s to identify chemically modified peptides, an analytical method that ionizes samples, separates them by ionic mass charge ratio, and measures the abundance of ions. The process begins with the enrichment of proteins using specific antibodies, with sodium dodecyl sulfate-polyacrylamide gel electrophoresis (SDS-PAGE) serving as the final step of preparation; The target protein on the gel is then isolated and digested with endonuclease; Finally, PTMs was characterized and analyzed by LC-MS/MS [24, 25]. The protein regulatory network shows remarkable diversity, including over 600 identified types of PTMs. The well-established classic modifications include phosphorylation, methylation, acetylation, ubiquitination, and SUMOylation. Additionally, specific cysteine modifications such as oxidation, nitrosylation, glutathionylation, and sulfhydration significantly contribute to protein regulation. Furthermore, glycosylation plays a prominent role in modifying numerous membrane proteins including O-GlcNAc and N-GlcNAc [10]. Recently, acylation modifications such as lactylation, butyrylation, succinylation, 3-hydroxybutyrylation, and propionylation, have also been found to occur in protein lysine residues [26–28].

Most classical PTMs function in the regulation of cellular energy metabolism, lipid metabolism, ketone body metabolism, and amino acid metabolism. Moreover, the PTMs such as methylation and acetylation also mediate the functions of nucleosome proteins, which are critical regulators of DNA damage repair, chromosome stability, and epigenetic inheritance. Additionally, glycosylation modifications are identified in proteins distributed in cell membranes, which play roles in cell protection, stability, and barrier.

The types and roles of PTM in tumor progression are broad. For example, protein phosphorylation is prevalent in many cancers. A typical example is phosphorylation at Ser37 of Pyruvate kinase 2(PKM2) in triple-negative breast cancer (TNBC), which is a biomarker for TNBC. Apostolidi et al. found that cyclin-dependent kinase (CDK) inhibitors and PKM activators can bind to Ser37 and affect the protein structure at the phosphorylation site, which can effectively inhibit cancer progression [29]. Recently, Peng et al. also found that PKM activators can play a similar therapeutic role in colorectal cancer [30]. Another typical example is the phosphorylation of the key molecule mTOR. Different kinases lead to different types of activation of mTOR, thereby activating different substrate proteins, including AKT, S6K, p53, etc., which has great potential value in the discussion of the development process of cancer [31]. Ubiquitination is involved in the development of cancer by promoting protein degradation. Ubiquitin-specific protease 7 (USP7) is a deubiquitinase that can deubiquitinate substrates by binding to them. Mouse double minute 2 homolog (MDM2) is a typical USP7 substrate, and specific USP7 inhibitors can promote the degradation of MDM2 by inhibiting the function of USP7, and ultimately activate the p53 pathway. This small molecule inhibitor has shown promising clinical performance [32]. In addition, proteolytic targeting chimera (PROTAC) technology is becoming increasingly mature. This technology links an E3 ubiquitin ligase ligand to a target protein-ligand through a linker, thereby bringing them into proximity and facilitating target protein degradation via the ubiquitin-proteasome pathway [33]. This approach can significantly enhance the druggability of difficult drug targets, making it crucial to advance the improvement and development of PROTAC drug technology. For example, Specific small ubiquitin-like modifier proteases 1 (SENP1), a deSUMOylase, can effectively deSUMOylate c-Myc and reduce c-Myc polyubiquitination and promote monoubiquitination. Finally, it enhances the stability of oncoprotein c-Myc. SENP1 is highly expressed in a variety of cancers, which has a broad stabilizing effect on c-Myc [34]. This study goes against the conventional wisdom that SUMOylation can stabilize proteins, it is possible that SUMOylation also could promote protein degradation, which requires more data to support it. In summary, we can roughly divide the role of PTM in cancer into two broad categories. On the one hand, in cancer, PTM on target proteins can directly regulate cancer signaling pathways. For example, the enzyme proteins of the glycometabolic pathway or lipid metabolic pathway have different activities before and after PTM. PKM2 is highly O-GlcNAc modified, which disrupts the stability of PKM2 tetramer and promotes PKM2 nuclear translocation. This modification is indispensable for inducing metabolic reprogramming of tumor cells and Warburg effect [35]. Glutaminase is another important metabolic enzyme. Wang et al. found that HDAC4 can effectively deacetylate glutaminase, which accelerates the catalytic decomposition of glutamine to glutamate, promotes the TCA pathway of tumor cells, and stimulate tumor metabolism [36]. In addition, acetyl-coA acetyltransferase 1(ACAT1) is a fatty acid metabolizing enzyme, and Tyr407 phosphorylation of ACAT1 promotes the stability of its tetramer morphology and accelerates tumor metabolism [37]. However, on the other hand, PTM of immune regulatory molecules can regulate tumor development by controlling tumor immune evasion or autoimmunity. The most typical example is that PD-1 has a variety of post-translational modifications: glycosylation, phosphorylation, and ubiquitination. PTM therapy targeting PD-1 in T cells may have a good effect of enhancing anti-tumor immunity [38].

In recent years, several reports have described promising therapeutic strategies for targeting changes in the level of post-translational modification of proteins in cancer signaling pathways, such as phosphorylated proteomics techniques used to uncover breast cancer patient populations characterized by phosphokinase activity, which may provide precision medicine and personalized treatment options [39]. Li et al analyzed the protein and phosphorylation levels of 10 tumors (including pancreatic cancer, non-small cell lung cancer, breast cancer, etc.) using proteomic phosphorylation technology, and analyzed the regulation of PTM in pan-cancer with large-scale multi-omics data by drawing multi-omics molecular maps [40]. The ability of multi-omics technology to link PTM to cancer occurrence and progression provides an effective way to expand our understanding of cancer and reveal the driving factors of cancer. Based on the important role of PTM in cancer and the lack of summary of PTM in the HNRNP family in previous studies, this review will summarize the association of the HNRNP family with cancers and mainly elucidate the underlying mechanisms of HNRNPs mediated by PTMs, as well as their potential implications in cancer therapy.

### Regulation of HNRNPs by PTMs

HNRNP A/B (A0, A1, A2/B1, A3)

The *HNRNPA/B* family is composed of *A0*, *A1*, *A2/B1*, *A3* and usually contains two RRMs and a long glycine-rich chain at the C-terminus. The phosphorylation in *HNRNP A0* at Ser84, which is activated by mitogen-activated protein kinase-activated protein kinase 2 (MAPKAPK-2) in mammalian macrophages. The phosphorylated *A* protein specifically binds to the AU-rich elements (AREs) of mRNA, thereby facilitating post-transcriptional regulation of key macrophage cytokines including Tumor Necrosis Factor α (TNF-α), cyclooxygenase-2 (COX-2) and macrophage inflammatory protein-1 (MIP-2) [41]. Cannell et al. found that the phosphorylated *HNRNP A0* at Ser84 activated by MAPKAPK-2 showed more apparent cytoplasmic localization compared to the non-phosphorylated *HNRNP A0* in lung cancer, and increased the stability of mRNA p27/growth arrest and DNA damage-inducible α (p27/Gadd45α) when DNA was impaired. In addition, the efficiency of platinum-based chemotherapy in lung cancer mouse model was enhanced through phosphorylation of *HNRNP A0* by MAPKAPK-2 [42]. (Table 1A).

**Table 1:** A summary table of the HNRNP family.

A							
	Site	Domain	Kinase	disease	Function	Target	Ref.
*A0*	Ser84-P	RRM	MAPKAPK2	NSCLC	Response to DNA damage Translational regulation mRNA stability	p27kip1 Gadd45α	[42]
*A1*	Lys3-Ub	N-terminal	TRAF6	CML	Splicing	Arhgap1	[17]
*A1*	Ser4-P Ser6-P	N-terminal	S6K2	CRC BC	Translational regulation mRNA transportation Splicing	Bcl-xL, xIAP PKM	[44, 45]
*A1*	Lys8-Ub	N-terminal	ZFP91	HCC	Splicing	PKM	[50]
*A1*	Lys113-SUMO	RRM	SAE2 UCB9	PDAC BLCA	Lymphangiogenesis	KRAS ESCRT	[47, 199]
*A1*	Ser199-P Arg218/225-Me	RGG	Akt PRMT5	GBM	IRES-trans-acting factors activity	Cyclin D1 c-MYC	[48, 49]
*A2/B1*	Lys274/305-Ub	—	VHLα	RCC	Degradation	c-MYC	[59]
*A2/B1*	Lys108-SUMO	RRM	UCB9	GBM	Exosomal packaging and sorting	STAT3	[56]
*A2/B1*	Lys108-SUMO	RRM	UCB9	NSCLC	Lymphangiogenesis, lymphatic metastasis	PROX1	[57]
*A2/B1*	Lys27-Ub Lys48-Ub	RRM	FBXO11	HCC	Degradation	FASN ACC1	[58]
*C*	Tyr57-P	RRM	uPA	NSCLC	mRNA stability	uPAR	[68]
*CL*	Lys175-SUMO	—	UBA2	GBM	Translational regulation	FOXD1	[72]
*D*	Ser83/87-P Thr91-P	—	mTORC2	—	Phosphorylate Akt mRNA stability mRNA binding	Akt	[75–77]
*D*	Arg-Me (*4) (in the RGG)	RGG	—	—	Maintain RGG activity	VEGF	[79]
*E1*	Ser43-P	KH1	Akt	NSCLC BC	Promote the EMT	TGF-β	[200]
*E1*	Thr60/127-P	KH1/KH2	Pak1	BC	mRNA binding Translational regulation Splicing	Caper-α	[18]
*E2*	Ser173/189-P, Ser272-P, Thr213-P	—	MAPK^ERK1/2^	CML	mRNA binding Translational regulation	C/EBPα G-CSFR	[91]
*F*	Lys98-Ac/Ub, Lys224-Ac/Ub	RRM	—	BC	Splicing	Bcl-x	[19]
*G*	Thr216-P	SPRY	TAK1	—	—	—	[101]
*I*	Ser16-P	NLS	PKA	—	Nucleoplasmic transport Localization	—	[111]
*I*	Thr138-P	RRM	Lkb1	tumorigenesis	Splicing	Scf	[112]

B							
	Site	Domain	Kinase	disease	Function	Target	Ref.
*K*	Cys132-Redox	—	—	tumorigenesis	Transcriptional Inhibit	HSF1	[146, 147]
*K*	Ser116-P	—	Akt	PCa	mRNA binding	AR	[149, 201]
*K*	Ser116 Thr120 related	—	FBXW7	PDAC	Recognizing and binding	miR-223	[202]
*K*	Lys63-Ub	KH1	SCF^Fbxo4^	SKCM	mRNA binding	c-MYC	[140]
*K*	Ser284/353-P	KNS	MAPK^ERK1/2^	NPC	mRNA stability Localization	—	[143–145]
*K*	Lys422-SUMO	KH3	Pc2 PIAS3	CRC	HNRNPK-p53-p21	p53	150–152]
*K*	Arg296/299-Me Ser302-P	—	—	—	Cell Apoptosis	—	[153]
*K*	Arg296/299-Me Ser379-P	—	PRMT1	TNBC	Cell Migration	β-catenin	[13]
*K*	Tyr458-P	C-terminal	c-Src	—	mRNA binding	DICE	[203]
*L*	Ser52-P	—	AKT	NSCLC	mRNA binding, Splicing	caspase-9	[118, 119]
*L*	Tyr359-P	—	Anaerobic	tumorigenesis	Splicing, Localization	VEGFA	[120–122]
*L*	Ser544-P	RRM	CaMkIV	tumorigenesis	mRNA binding	CaRRe	[123, 124]
*L*	Ub	—	FBXO16	OV	degradation	—	[125]
*M*	Ser574-P	—	P38/MAPK	Innate immunity	mRNA binding	IL-6	[159]
*P*	Tyr6/296-P	QGSY/NES	EGFR	fibrotic diseases	Localization Transcriptional regulation	collagenVI	[161]
*P*	Ser182/183-P	RGG	casein kinase1δ/ε	ALS AD PD	Solubility of proteins	—	[162]
*P*	Ser256-P	RGG	PKCβII	CML	Protein stability	—	[165]
*P*	Lys315/316-Ac, Lys510-Ac	RRM, NLS	CBP(P300)	ALS	mRNA binding Localization	— Transportin1	[166]
*P*	Lys333-SUMO	RRM	UBC9	GBM	Protein stability	ATG4D	[167]
*P*	Arg-Me	NLS	PRMT1	—	Localization	—	[168]
*P*	Tyr526-P	C-terminal	Abl	—	Localization	—	[169]
*Q*	—						
*R*	—						
*U*	Ser59-P	—	DNA-PK PLK1	—	Response to DNA damage Chromosome alignment Chromosome segregation	—	[177–179]

This table contains the types of post-translational modifications, the sites where the post-translational modifications occur, and the domains, the associated cancer or disease types, functions, and downstream targets. —: No progress or results.

Similar to *A0*, *HNRNP A1* exhibits a high affinity for AREs [43]. The ubiquitination of Lys3 in *A1* mediates the alternative splicing of Arhgap1. This process has been proven to promote hematopoietic function in mouse models, suggesting a promising target for the treatment of myeloid malignancy [17]. Phosphorylation at Ser4/6 residue of the *A1* protein, which is activated by S6K2 kinase in colorectal cancer, controls the cytoplasmic transport and translation of BCL-x(L) and x-linked inhibitor of apoptosis (XIAP). Furthermore, phosphorylation at Ser6 can modulate PKM splicing, thereby exerting an impact on glucose metabolism reprogramming in cancer cells. The mRNA binding ability of *A1* protein is jointly controlled by phosphorylation modifications at Ser4/6 and SUMOylation at the Lys183 [44, 45]. SAE2-mediated SUMOylation of *A1*-Lys113 in pancreatic cancer holds promise as a potential therapeutic target for KRAS-associated lymph node metastasis. Meanwhile, SUMOylation of Lys113, based on Ubc9 activation in bladder cancer, facilitates the recognition of LncRNA-ELNAT1 by the endosomal sorting complex required for transport (ESCRT) and promotes its packaging into extracellular vesicles (EVs), which ultimately induces lymphatic vessel assembly and generation [46, 47]. In glioblastoma, the phosphorylation of *HNRNP A1* at Ser199 hampers the function of the IRES trans-acting factor (ITAF) on cyclin D1 and c-myc mRNA, while the methylation of *HNRNP A1* Arg218/225 sustains internal ribosome entry site-binding (IRES-binding) and preserves cyclin D1 or c-MYC IRES activity. The mutual influence of both *HNRNP A1* Ser199 phosphorylation and *HNRNP A1* Arg218/225 methylation establishes a regulatory network [48, 49]. Zinc finger protein 91 (ZFP91) is an E3 ligase. Chen et al reported that ZFP91 can promote ubiquitination of *HNRNP A1* at Lys8, which leads to degradation of *A1* protein and down-regulation of its expression in HCC cells. The mechanism is to inhibit metabolic reprogramming, proliferation, and migration of hepatocellular carcinoma through alternative splicing of PKM [50].

The *HNRNP A3* interacts with the cis-acting element A2 recognition element (A2RE) and participates in the transport of A2RE-related mRNA. Importantly, *HNRNP A3* exhibits the capability of shuttling between the nucleus and cytoplasm [51]. However, the precise epigenetic mechanisms underlying the role of *HNRNP A3* in cancer remain relatively unexplored, providing significant opportunities for further comprehensive studies.

In the *HNRNP A/B* subfamily, *HNRNP A2/B1* binds to A2RE to induce subcellular localization of various pre-mRNAs within the cell. The translation products of these pre-mRNAs include myelin basic protein (MBP), myelin-associated oligodendrocyte basic protein (MOBP), and microtubule associated protein (MAP), which facilitates the translocation of pre-mRNAs from the nucleus to the cytoplasm and promotes their translation [52, 53]. SUMOylation of *A2/B1* is a decisive regulator by facilitating the selective packaging of miRNAs into exosomes and indicates how miRNAs are integrated into exosomes and exported to cells to produce remote regulation [54, 55]. For example, recent studies by Guo et al have shown that SUMOylation of *HNRNP A2/B1* can promote the exosomal packaging and sorting pathway of miR-204-3p under hypoxia conditions. Through the signal transducer and activator of transcription 3 (STAT3) pathway, angiogenesis in the microenvironment of glioblastoma is promoted, thereby alleviating the survival pressure of cancer cells under hypoxia conditions and deepening the deterioration of cancer cells [56]. In non-small cell lung cancer, circTLCD4-RWDD3, a circular RNA, binds to *HNRNP A2/B1* and promotes SUMOylation of the Lys108 of *HNRNP A2/B1* by ubiquitin-conjugating enzyme 9 (UBC9). And then, the SUMO interaction motif (SIM) in ALG-2-Interacting Protein X (ALIX) identified the recruitment program that activates ESCRT-III after SUMOylation. Ev-packaged circTLCD4-RWDD3 is taken up by human lymphatic endothelial cells to promote the transcription of lymphangiogenesis factor Prospero homeobox 1 (PROX1) and promote lymphangiogenesis and lymphatic metastasis in non-small cell lung cancer [57]. Zhang et al. found that the cullin-associated and neddylation dissociated 1 (CAND1), which has an abnormally high expression level in liver cancer cells, can inhibit F-box protein 11 (FBXO11) activated *HNRNP A2/B1* ubiquitination, thereby inhibiting the degradation of *HNRNP A2/B1*. Therefore, targeting the CAND1-SCF^FBXO11^-*HNRNP A2/B1* axis may be a potential therapeutic strategy for liver cancer [58]. Notably, in renal cell carcinoma, the ubiquitination in *HNRNP A2/B1* at Lys274/305 residues is regulated by von hippel-lindau α (VHLα). Additionally, *HNRNP A2/B1* also mediates the alternative splicing process of VHL. These findings suggest a potential feedback loop involving VHLα-*HNRNP A2/B1* may be involved in the anti-tumor mechanisms [59]. The recent investigations have unveiled that *HNRNP A2/B1* can specifically interact with m6A [60], which can interact with MIR100HG and control the activity of the Wnt signaling pathway through recognition of m6A sites on transcription factor-7-like-2 (TCF7L2) mRNA in colorectal cancer [61].

### HNRNPC, D, and E

Apart from *HNRNP A2/B1*, another core particle responsible for 40S *HNRNP* nucleation is *HNRNP C* [62]. The coding region of the *HNRNP C* gene contains a NLS at positions 155-161 amino acids, flanked by an upstream RRM and a downstream basic zipper-like leucine motif (bZLM) [63, 64]. The *HNRNP C* regulates the ability of translation in a stability- and cell cycle-dependent manner by interacting with the poly-U tail located within the 3′ and 5′-UTR of the mRNA [65, 66]. Moreover, *HNRNP C* categorizes nascent transcripts according to their length and subsequently facilitates their transportation to the cytoplasm [67]. Recent researches by Sayaka Dantsuji et al. indicate that *HNRNP C* interacts with the cap-binding complex (CBC) to enhance the binding of CBC to transcription and export (TREX) and weakens the binding of CBC to phosphorylated adapter for RNA export (PHAX), which facilitates the nuclear-cytoplasmic transport of the transcripts exceeding 300 nucleotides [64].

In terms of protein modifications, uPA-mediated phosphorylation in *HNRNP C* Tyr57 could stabilize urokinase-type plasminogen activator receptor (uPAR) mRNA in lung cancer and is essential for facilitating the interaction between the *C* protein and the 3’UTR region [68]. When cells are stimulated with low concentrations of H_2_O_2_, which can induce cellular oxidative stress, the Ser225-Ser228 and Ser240 residues in *HNRNP C* can be rapidly phosphorylated. However, the functional and phenotypic meanings of phosphorylation at these residues in cancers remain to be studied in depth [69, 70]. The *HNRNP CL* is a branch of the *HNRNP C* subfamily, and their amino acid sequence and structure are highly similar. Recently, Cao et al. interpreted SUMOylation of *HNRNP CL2* to promote the progression of glioblastoma. The SUMOylation of Lys175 in *HNRNP CL2* is facilitated by Ubiquitin-like modifier-activating enzyme 2 (UBA2, a subunit of E1). The SUMOylation of *HNRNP CL2* enhances its stability, and it is subsequently anchored to forkhead box D1 (FOXD1) mRNA to promote FOXD1 expression. FOXD1, an oncogene upregulated in cancers [71], promotes the transcriptional expression of DKK1 and malignant proliferation and migration of glioma cells [72].

Similar to the *HNRNP A/B* family, *HNRNP D* also consists of two RRMs, a C-terminal glycine-rich region, and an RGG box. The similarity between *HNRNP D* and *HNRNP A0* is that both of them have specific affinity for AREs [73]. Further investigation of *HNRNP D* showed that it has four distinct isoforms: p37, p40, p42, and p45, and each of them exhibits unique functions that mediate different phenotypic outcomes [74]. In these isoforms, only p40 and p45 of *HNRNP D* possess phosphorylation at three specific amino acid residues: Ser83, Ser87, and Thr91. The selective phosphorylation at these sites significantly modulates the binding affinity of *HNRNP D* with ARE elements [75, 76]. The *HNRNP D*, especially p40 and p45 isoforms, could promote Akt phosphorylation at Thr308, Thr450, and Ser473. Meanwhile, mTORC2 can activate the AKT-mTOR pathway and induce *HNRNP D* phosphorylation [77]. Furthermore, ubiquitin-induced degradation of the p40 isoform is dependent on autophosphorylation of Ser83 and Ser87 [78]. Additionally, LC-MS/MS analysis revealed the existence of four methylated Arg residues in the C-terminal RGG box of *HNRNP D*, which play a crucial role in maintaining the functional activity of *HNRNP D* RGG box, thereby leading to the inhibition of vascular endothelial growth factor (VEGF) expression [79].

The *HNRNP E* comprises at least four isoforms known as Poly(rC)-binding protein (*PCBP*), namely *E1* to *E4* [80]. All four isoforms have three typical type I KH and contain a GXXG loop. However, Only *E1* and E2 were expressed stably in various cells and tissues, while *E3* and *E4* showed a trend of low expression in some tissues or developmental stages [81, 82]. Notably, E3 and E4 have not been classified as part of the *HNRNP* family due to their localization in the cytoplasm [83]. The phosphorylation of *PCBP1* at Ser43 is activated by Akt2 kinase, which drives TGF-β-induced epithelial to Epithelial-Mesenchymal Transition (EMT) in NSCLC and breast cancer [84, 85]. In the cytoplasm, phosphorylation of *PCBP1* at Thr60 and Thr127 are activated by Pak1, reduces its mRNA binding affinity, thereby leading to the mRNA translation suppression. In the nucleus, *PCBP1* phosphorylation at Thr60 and Thr127 not only governs transcriptional regulation by binding to specific gene promoters but also controls the alternative splicing process of Caper α (a U2 snRNP auxiliary factor-related protein) [18].

Furthermore, the *PCBP1* protein is widely recognized as a tumor suppressor. For instance, depletion of *PCBP1* leads to activation of phosphatase of regenerating liver-3 (PRL-3) and the AKT signaling pathway, thereby promoting tumorigenesis. Moreover, *PCBP1* binds to the 3’UTR region of P27 transcript through its KH1 domain, which promotes the stability of P27 and inhibits tumorigenesis as well as cell cycle progression [86]. However, the *PCBP2* has dual function in cancer. Janecki et al. found that *PCBP2* promotes the stability and translation efficiency of p53-mRNA by binding to the p53 transcript, and inhibits the progression of colorectal cancer [87]. On the contrary, in gastric cancer, *PCBP2* interacts with the 3′UTR region of CDK2 transcript, thereby accelerating the cell cycle of gastric cancer cells and promoting tumorigenesis. Moreover, the oncogenic role of *PCBP2* has been verified in various malignancies including glioblastoma and breast cancer [88–90].

Alterations in the tyrosine kinase activity of the p210-BCR/ABL oncoprotein drive the development of chronic myeloid lymphoma (CML). Moreover, upregulation of p210-BCR/ABL expression is a prerequisite for *PCBP2*-induced anti-differentiation signals. BCR/ABL and MAPK^ERK1/2^ kinases can activate *PCBP2*, leading to phosphorylation at Ser173, Ser189, Ser272, and Thr213. The phosphorylation in these residues increases the stability of *PCBP2* protein and enhances its affinity for the 5′UTR of CCAAT/enhancer-binding protein α (C/EBPα) mRNA. After deletion of *PCBP2* phosphorylation, this affinity is also reduced, which promotes C/EBPα expression efficiency and G-CSFR-driven neutrophil maturation, initiating differentiation of chronic myeloid leukemia blastoblastic crisis (CML-BC) progenitor cells [91].

### HNRNPF and H

**Fig. 2:**
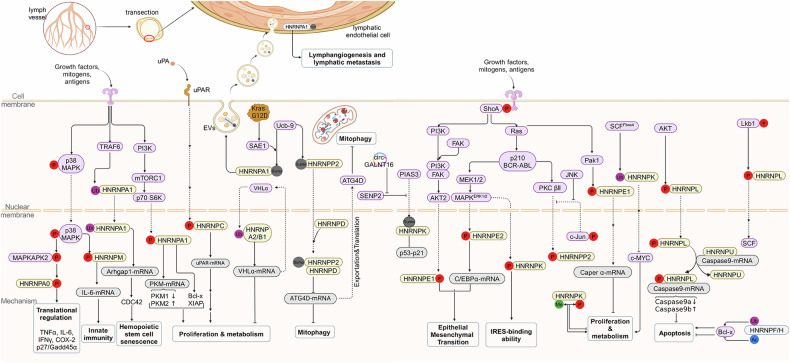
Mechanisms of action of the HNRNP family in cancer (Created with BioRender.com). The top of this picture represents the extracellular environment. From the top to bottom are the cell membrane, the cytoplasmic matrix, and the nucleus. At the bottom are the mechanisms affected by the HNRNPs. The proteins in purple are kinases or upstream regulatory enzymes, HNRNPs are in light blue, downstream transcripts are in gray, and the final mechanism is in black boxes. P Phosphorylation; AC Acetylation; Me Methylation; Ub Ubiquitination; SUMO Sumoylation.

The *HNRNP F* and *HNRNP H* exhibit a significant structural similarity, and both of them harbor three qRRMs and two glycine-rich domains. The RRMs of *HNRNP F* and *HNRNP H* are not completely conserved, which are classified as qRRMs [92]. Currently, the *HNRNP F/H* family consists of *HNRNP F*, *HNRNP H1*, *HNRNP H2*, and G-rich RNA sequence binding factor 1 (*GRSF1*). Subcellular localization analyses have revealed that *HNRNP F*, *HNRNP H1*, and *HNRNP H2* mainly localize in nucleus, while *GRSF1* localizes to the mitochondria. *GRSF1* possesses a unique glycine-rich region in its N-terminal structure, which may contribute to its mitochondrial localization and gene regulation mediated by *GRSF1* [93]. The specific crosstalk between acetylation and ubiquitination (KAc/Ub) in *HNRNP F* at Lys98 and Lys224 is especially important for Bcl-x(L)/x(S) splicing control in breast cancer. This crosstalk was also found at residue Lys167 in *HNRNP H* [19] (Fig. 2).

### HNRNPG, I, and L

Only *HNRNP G* in *HNRNP*s was confirmed to be targeted by glycosylation modification, which was designated as RNA-binding motif protein X-linked (*RBMX*) due to the location of its open reading frame (ORF) on X chromosome. Similarly, the homologous counterpart on the Y chromosome is called *RBMY* [94, 95]. In developmental and biological investigations related to *RBMY*, it has been established that *RBMY* plays a crucial role in the ontogeny and viability of males [96]. The primary structures of *RBMX* and *RBMY* show a high degree of homology, and both of them contain a single RNA recognition motif (RRM). In comparison to *RBMX*, the downstream sequences in *RBMY* include four repeated copies of serine-arginine-glycine-tyrosine (SRGY) motifs, while *RBMX* only holds one copy of the SRGY motif. *RBMX* is strongly associated with mitosis, and it has been shown that lacking *RBMX* exhibits significant impairment in sister chromatid cohesion and homologous chromosome segregation and results in mitotic disruption [97, 98]. Several studies have shown that the *RBMX* gene is a tumor suppressor in many cancers, including endometrial cancer and papillary thyroid cancer [99, 100]. In a recent report, the Ser216 residue of *RBMX* was identified as a phosphorylation substrate for TGF-β activated kinase 1 (TAK1), however, its precise role in cancer has not been reported [101]. Interestingly, *RBMY*, the predicted homolog of *RBMX*, has been identified as a potential driver gene for HCC in males [102]. Recent research has confirmed that the PIM1 kinase is responsible for the phosphorylation of *RBMY*, and the cytoplasmic accumulation of phosphorylated *RBMY* promotes HCC metastasis [103].

The *HNRNP I*, also known as polypyrimidine tract binding protein 1 (*PTBP1*), contains four RRMs, and these four RRMs in *HNRNP I* show significant similarity to the RRMs in *HNRNP L*. Moreover, *HNRNP L* exhibits significant homology with *HNRNP I* [104]. Distinguished from other nucleocytoplasmic shuttling *HNRNP*s, the shuttling of *HNRNP I* from nucleus to cytoplasm is independent from mRNA binding, which depends on its N-terminal NS domain to participate in the translation of mRNA in the nucleoplasm [104–106]. At the post-transcriptional level, *PTBP1* is downregulated in response to cellular oxidative stress, which induces alternative splicing of soluble guanylyl cyclase (sGC) and dimerizes *PTBP1* for degrading [107]. In addition, *PTBP1* modulates the stability of the myeloid cell leukemia 1 (MCL1) transcript and promotes the accumulation of MCL1 in the cytoplasm, which inhibits the apoptosis of tumor cells [108]. The *PTBP1* downregulation can increase the expression level of cell division cycle 42-v2 (CDC42-v2), which consequently inhibits tumorigenesis [109]. Moreover, *PTBP1* can repress the expression of autophagy-related genes (ATGs), thereby facilitating tumor growth and metastasis in colorectal cancer [110].

Protein kinase A (PKA) induces the phosphorylation modification of endogenous *HNRNP I* at Ser16 residue, which located in the NS domain of the primary structure, and Ser16 phosphorylation promoted the accumulation of *HNRNP I* in cytoplasm. On the contrary, the transport of non-phosphorylated *HNRNP I* from the nucleus to the cytoplasm was greatly reduced, leading to its nuclear accumulation [111]. In wild-type endothelial cells, Lkb1 activates phosphorylation of Thr138 residues of *PTBP1*. Dephosphorylation of *PTBP1* Thr138 leads to the removal of Scf exon 6, resulting in decreased Scf secretion. At the same time, phosphorylated *PTBP1* is destroyed to rescue the differentiation process of classical dendritic cells (DCs) generated from macrophage-DC precursors (MDPs) through common DC precursor (CDP), and maintains DC count to inhibit tumorigenesis [112].

The four highly conserved RRMs in *HNRNP L* are indispensable for splicing regulation. The *HNRNP L* binds to nascent transcripts and inhibits the inclusion of exons. The *HNRNP L* contains a glycine-rich sequence in the N-terminal and a proline-rich sequence between RRM2 and RRM3 [113]. Previous studies have shown that *HNRNP L*, *HNRNP I*, and *HNRNP E2* regulate splicing by binding to ESS. Notably, *HNRNP L*, a potent splicing repressor, exhibits stronger binding to ESS than the other two proteins [114]. The regulation of splicing is through the exon or intron retention [114, 115]. The *HNRNP L* is indispensable for the activation of H3 histone Lys36 methylation [116]. In NSCLC, *HNRNP L* is a potential suppressor of the p53 pathway, and *HNRNP L* silence induces the apoptosis in lung cancer cells by mediating the function of p53 [117]. Furthermore, Akt-mediated phosphorylation of *HNRNP L* at Ser52 hinders its interaction with *HNRNP U* on caspase-9 mRNA, thereby modulating the ratio of caspase-9a to caspase-9b splice variants, which inhibits cell apoptosis and promotes NSCLC progression [118, 119]. Phosphorylation at Tyr359 in *HNRNP L* is activated under hypoxia conditions, which promotes the development and progression of malignant tumor. Normally, *HNRNP L* predominantly localizes in the nucleus. Excessive accumulation of phosphorylated *HNRNP L* in the cytoplasm promotes VEGFA expression by weakening the binding of miR-297 with VEGFA, which leads to the increase of angiogenesis and leukocyte. Finally, there will be an increase in chronic inflammatory diseases, various cancers, or atherosclerosis [120–122]. Furthermore, the calcium signaling pathway is implicated in promoting tumor development. In this review, calcium/calmodulin-dependent protein Kinase IV (CaMKIV) phosphorylates the Ser544 residue of *HNRNP L*, which augments the affinity for the CaRRE element at the 3′ splice site of mRNA [123, 124]. F-box protein 16 (FBXO16) is an E3 ubiquitin ligase that can promote ubiquitin-proteasome degradation of *L* protein through ubiquitination of *HNRNP L*, and ultimately prevent the malignant development of ovarian cancer [125].

### HNRNPK

KH homeodomains were initially identified in *HNRNP K*, which include three KH domains that primarily recognize and bind to specific RNA and DNA molecules [126, 127]. A classical NLS sequence is located upstream of KH1, which could maintain the stability and cellular localization of *HNRNP K* [128]. Additionally, a nucleo-cytoplasmic shuttling domain, also known as KNS, has been identified between the KH2 and KH3 regions, which is similar to the NS [129]. The *HNRNP K* specifically binds to polyC-rich or cytosine-rich pre-mRNAs, which enables the precise splicing of pre-mRNA transcripts [130]. Currently, many studies have shown that the overexpression and abnormal cytoplasmic localization of *HNRNP K* are potential prognostic indicators for multiple malignancies [131–135]. In terms of transcriptional regulation, *HNRNP K* was able to bind to the CT element region within the c-MYC gene promoter, which promotes recruitment of RNA polymerase II and enhances transcription of the c-MYC gene. Both *HNRNP K* and *HNRNP E1/E2* can motivate ribosomal site activity, thereby promoting translation of c-MYC mRNA [136, 137]. However, deletion of chromosome 9 results in the loss of one single copy of the *HNRNP K* gene and plays a carcinogenic role by inhibiting p53-dependent p21 expression and promoting tumor development in patients with hematologic malignancies, which indicates that *HNRNP K* shows oncogenicity not only in various solid tumor but also in hematologic malignancies such as leukemia [138].

The Skp1-Cullin-1-F-box (SCF) E3 ligase primarily participates in the process of ubiquitin degradation. The Fbox family is a core component of SCF E3 ligases and substrate recognition molecules [139]. In melanoma, the Fbxo4 component of the SCF E3 ligase mediates the K63 ubiquitination of *HNRNP K* to suppress the activity of *K* protein, which could suppress cancer cell migration and proliferation. Moreover, the SCF^Fbxo4^-*HNRNP K*-c-Myc axis controls the carcinogenicity and mRNA binding capacity of *HNRNP K* [140]. (Table 1B)S100B, a member of the acidic Ca^2+^-binding protein family, could regulate many cellular processes including cell proliferation, differentiation, Ca^2+^ homeostasis, protein phosphorylation, and other essential signaling pathways [141]. S100B can promote the cytoplasmic localization of *HNRNP K*. Natarajan et al. constructed a THP1-*HNRNP K*^K219I/S284D/353D^ model in normal human monocytes, which was a model without *HNRNP K* Lys219 methylation and Ser284/Ser353 phosphorylation. With this model, they found S100B-mediated cytoplasmic translocation of *HNRNP K* [142]. However, it is not known whether this feature has an inhibitory effect on cancer development in malignant cells. In nasopharyngeal carcinoma and colorectal cancer, phosphorylation of *HNRNP K* at Ser284 and Ser353 by MAPK^ERK1/2^ kinase enhances the stability of the *K* protein and facilitates cytoplasmic transport of *HNRNP K* [143–145].

Heat shock factor 1 (HSF1) is a transcription factor that is activated in cells under heat shock conditions or related stresses, which specifically binds with heat shock elements (HSE) located in nucleus. LC-MS/MS identification indicates that Cys132 of *HNRNP K* contains thiol oxidation modification, which enhances the interaction between *HNRNP K* and HSF1 and inhibits the binding of HSF1 with HSE [146]. HSF1-targeting has long been a therapeutic strategy for neurodegenerative diseases and various cancers. Therefore, studying the inhibitory mechanism of *HNRNP K* could potentially contribute to the development of anticancer drugs [146, 147].

Cyproterone acetate (CPA), a steroidal anti-androgen with progestin-like activity, is commonly used in the clinical treatment of advanced prostate cancer. In vivo, CPA mediates AKT hyperphosphorylation of *HNRNP K* at Ser116 and increases its tight binding to androgen receptor (AR), which delays prostate cancer progression [148, 149].

Circ-GALNT16 is associated with a good prognosis of colorectal cancer. It can enhance the transcription and expression of the *HNRNP K*-p53-p21 axis by binding to the KH3 domain of *HNRNP K* and promoting SUMOylation at the Lys422 site in colorectal cancer [150]. This study is similar to many previous studies depicting the effect of K422-SUMOylation on DNA damage repair [151, 152]. The methylation of *HNRNP K* at Arg296/299 could inhibit the phosphorylation of *HNRNP K* at Ser302 mediated by PKCdelta, and thus, PKC-mediated apoptosis is inhibited [153], which has significant implications for understanding how cancer cells evade apoptosis. In triple-negative breast cancer, Aurora-A kinase phosphorylates *HNRNP K* at Ser379, inhibiting cancer cell migration. Meanwhile, the *HNRNP K* phosphorylation can be inhibited by Arg methylation in the KI region. However, when Ser379 phosphorylation was lost, the migration ability of cancer cells increased through *HNRNP K*-β-catenin-MMP12 axis accompanied by the *HNRNP K* Ser379 phosphorylation deletion [13]. Zhu et al. found that ubiquitination of *HNRNP K* is also associated with the β-catenin pathway. PROX1 is abnormally high expressed in breast cancer. PROX1 binds to *HNRNP K* and inhibits its ubiquitination, thereby promoting *K* protein stability and ultimately activating the *HNRNP K*-β-Catenin-Wnt axis to promote breast cancer development [154]. Han et al. discovered a novel lncRNA--syndecan-binding protein 2-antisense RNA 1 (SDCBP2-AS1), which can inhibit the SUMOylation and promote the ubiquitination of *HNRNP K* by binding *HNRNP K* in gastric cancer cells. After down-regulating SDCBP2-AS1 expression level in vitro, *HNRNP K* ubiquitination level decreased successively, and then promoted the activity of downstream target genes regulated by *HNRNP K*/β-catenin, leading to the development and metastasis of gastric cancer [155]. These studies suggest that *HNRNP K*/β-catenin may be a potential anti-tumor therapeutic target.

### HNRNPM, P, Q, R and U

There are three RRMs in the *HNRNP M* protein. The N-terminal of the protein contains a unique py-NLS sequence (NLS sequence ends with a conserved proline (P) and a tyrosine (Y)), which is distinct from the classical NLS sequence [156, 157]. The *M* protein shows a strong binding affinity to both poly-G and poly-U RNA homopolymers [158]. In innate immunity, *HNRNP M* binds to IL-6 mRNA and represses the splicing process. However, when Ser574 in the *M* protein is phosphorylated by P38-dependent MAPK, the splicing activity of *HNRNP M* is significantly weakened, which is beneficial for IL-6 responding to viral infection or clearance of cancer cells in innate immune process [159].

The *HNRNP P2*, also known as translocated in liposarcoma (*TLS*) or fused in sarcoma (*FUS*), contains a highly conserved Serine-Tyrosine-Glycine-Glutamine (SYGQ) motif at the N-terminal and single RRM. There is an NLS sequence in the C-terminus of *HNRNP P2*, and an NES sequence is included in the RRM. Moreover, *FUS* contains multiple RGG motifs and an RGG-ZnF-RGG motif in the C-terminal [160]. Epidermal growth factor receptor (EGFR) kinase phosphorylates two tyrosine sites in *FUS*, Tyr6 and Tyr296. Phosphorylation of these two sites facilitates the nuclear translocation of *FUS* and the interaction of *FUS* with integrin α-4, which may provide a potential new therapeutic target for fibrotic diseases [161]. Recently studies have shown that Casein kinase 1δ/ε exhibits the ability to phosphorylate many serine sites in *FUS*, especially Ser182 and Ser183. The phosphorylation of serine residues significantly strengthens the solubility of the *FUS* protein in patients with neurodegenerative disorders, which may have implications for targeted therapy of neurodegenerative diseases [162]. The c-Jun protein, a member of the Jun family, is an integral component of the transcription activator protein1 (AP-1) [163]. Normally, protein degradation or hydrolysis is facilitated by the ubiquitin-proteasome pathway, which is associated with the 26S proteasome [164]. However, *FUS* hydrolysis is induced by c-Jun rather than through the ubiquitin-proteasome degradation pathway and is enhanced by *HNRNP A1*. BCR-ABL, an antiapoptotic gene with high tyrosine kinase activity, enhances the stability of *FUS* through PKCβII-mediated phosphorylation of *FUS* at Ser256 [165]. Acetylation in *FUS* at Lys315, Lys316, and Lys510 activated by P300/ CBP plays a regulatory role in *FUS* subcellular localization and mRNA binding [166]. In glioblastoma, lncRNA-RMST enhances UBC9-mediated SUMOylation at Lys333, which significantly enhances the stability of the *FUS* protein. The interaction between SUMOylated *FUS* and *HNRNP D* promotes the stability of autophagy-related 4D cysteine peptidase (ATG4D), which inhibits mitochondrial autophagy and suppresses glioblastoma development [167]. The methylation of arginine in the C-terminal NLS of *FUS* could also control its nucleocytoplasmic transportation [168]. Similarly, ABL kinase activates the phosphorylation of the C-terminus Tyr526 residue, which promotes the nucleocytoplasmic transport of *FUS* [169]. Currently, the research focus of *HNRNP P2* is mainly in neurodegenerative diseases; however, it is noteworthy that *FUS* also shows oncogenic properties [162, 167]. Therefore, the role of *HNRNP P2* in cancer still has great potential to be studied.

The *HNRNP Q*, also known as *GRY-RBP*, *NSAP1*, or *SYNCRIP*, has an acidic amino acid in the N-terminal region, which has three consecutive RRMs, and one of them contains a NLS at the C terminus, which regulates the subcellular localization of the *Q* protein. In addition, the C terminus contains an RGG box [170]. At present, although the molecular mechanism of *HNRNP Q* regulated by PTMs in malignancies has not been accurately reported, the variation of *HNRNP Q* at the protein level is of great significance for the induction of neurological diseases such as autism spectrum disorders (ASD) and neurodevelopmental disorders (NDD) [171].

The *HNRNP R* is similar to *HNRNP Q*, which induces diseases such as neurodevelopmental disorders, while relevant studies in cancer are still insufficient [172]. Several tyrosine phosphorylated residues on *R* protein have been identified in sarcoma cell lines and human tumor cells [173], but the function of these modified residues remains to be studied.

The *HNRNP U*, also known as *SAF-A*, has at least 4 conserved structural domains: SAP, SPRY, AAA+, and RGG. In these domains, the SAP domain shows DNA-binding activity, the AAA+ domain drives various cellular activities mediated by ATPase, and the RGG domain is an RNA-binding domain and rich in Arg-Gly-Gly motifs [174–176]. The phosphorylation of protein *U* at Ser59 activated by DNA-dependent protein kinase (DNA-PK) can mediate DNA damage. Moreover, Ser59 is phosphorylated by polo-like kinase 1 (PLK1) rather than DNA-PK during mitosis, which promotes key biological processes such as chromosome alignment and segregation during cell division [177–179]. Additionally, *HNRNP U* is implicated in the regulation of PTMs of many specific proteins. For example, Kim et al. revealed that *HNRNP U* binds transcription factor IIH (TFIIH) and inhibits the TFIIH-mediated phosphorylation of the C-terminal domain (CTD) of Pol II [180].

## Functional prediction of novel PTMs in the HNRNPs

Recently, novel acylation modifications, including lactylation, butyrylation, succinylation, and 3-hydroxybutyrylation [26, 27], have been confirmed to be mapped to various proteins with a broad function distribution. In cancer cells, after identifying these novel modifications in members of the HNRNP family using LC-MS/MS technology, their potential function can be assessed. Firstly, it is possible to determine whether the amino acid residue at the modified site is evolutionarily conserved in multiple species because highly conserved positions are likely to be functionally significant. Subsequently, it is important to determine whether the modification site is located in a domain commonly found in the HNRNP family, such as RRM, qRRM, RGG, bZLM, or NLS, and the potential impact on protein structure or function could be roughly inferred, as well as alternative splicing or other signaling pathways that may be affected eventually. Finally, based on the speculation and hypothesis of its function, and combining the results of LC-MS/MS, we can classify it into two models: (1). The level of modification is significantly up-regulated or down-regulated. (2). The level of modification changes but is not significant. For the former, we can construct a cell model by first knocking out the endogenous target proteins in the cell, and then creating two cell models: one rescuing the wild-type and the other rescuing the modified site mutants. Subsequently, the function of this modification site in cancer can be explored using these cell models through in vitro experiments. Ultimately, the actual function and mechanism of this site can be elucidated, and its druggability and targetability can be verified. For proteins whose modification levels have changed but are not significant, experimenters could analyze their overall action network through bioinformatics methods and explore changes in the overall modification level in cancer, and finally explain the overall changes in the cancer microenvironment.

## The potential applications of HNRNPs PTMs in cancer therapy

The major limitations of traditional cancer treatments such as chemotherapy and radiation therapy are that they kill not only cancer cells but also normal cells in human body [181, 182], which has prompted extensive research efforts in developing targeted anti-cancer therapies that selectively attack cancer cells while sparing healthy ones. Therefore, it is more and more important to design targeted anti-tumor drugs and implement them in clinical treatments. We summarize the selected drugs targeting HNRNPs protein PTMs, some of which have advanced to clinical trials with promising results (Table 2). The value of a single agent for the activating enzymes of PTMs is gradually diminishing, and combination therapy has been necessary in clinical trials.

**Table 2:** A list of only a small number of tumor-targeting drugs related to the HNRNP family.

Drugs	Phase	Target	Tumor types	ClinicalTrials.gov Identifer
CI-1040	II	MEK	BC, NSCLC, PDAC	NCT00033384
Rapamycin	I/II	mTOR	OV, HCC	NCT05836025 NCT02724332
PP242	—	mTOR	GBM	—
Torin1; AKTi-1/2	—	mTOR	—	—
GSK3235025	—	PRMT5	GBM	—
TAK-981	I/II	SUMO-E1	Solid Tumors, RRRM, GBM	NCT05976334 NCT04776018
CPA	II/III/IV	AR	Adenocarcinoma, PC	NCT04925180 NCT04964193 NCT04848181

—: No progress or results.

*BC* breast cancer, *OV* ovarian cancer, *NSCLC* non-small cell lung cancer, *PDAC* pancreatic ductal adenocarcinoma, *HCC* hepatic cell carcinoma, *GBM* glioblastoma, *RRRM* relapsed and/or refractory multiple myeloma, *PC* prostate cancer.

### Targeting tyrosine kinases

Tyrosine kinases play a pivotal role in cellular signal transduction pathways, regulating multiple functions such as cell growth, differentiation, and apoptosis [183], which are broadly divided into two main groups: receptor tyrosine kinases (RTKs) and non-receptor tyrosine kinases (NRTKs). RTKs are localized mainly on cell membranes and perform functions by interacting with ligands. Such as vascular endothelial growth factor receptor (VEGFR) and epidermal growth factor receptor (EGFR) as we mentioned earlier. Conversely, most NRTKs are localized in the cytoplasm, such as Abl and Src kinases [184]. The activities of tyrosine kinases are associated with tumorigenesis and tumor progression, such as promoting cell proliferation, migration, and inhibiting apoptosis [183].

Tyrosine kinase inhibitors (Tkis) can inhibit tumor cell signal transduction by targeting tyrosine kinase activity. A previous example is that targeting MEK can inhibit cancer through *HNRNP* molecules and eventually target CML therapy.

Among the factors driving CML, upregulation of p210 BCR-ABL can activate the MEK-MAPKERK1/2-*HNRNP E2* (phosphorylation) pathway and promote the EMT process. CI-1040 is a MEK-targeted inhibitor. After treatment of CML cells with CI-1040, the expression of MAPKERK1/2 is inhibited, and then the binding of *HNRNP E2* and C/EBPmRNA is reduced by down-regulating the phosphorylation level of *HNRNP E2*, thus inhibiting the progression of CML [91].

In addition, Tkis that have been used in the clinic are relatively mature, and these Tkis include drugs such as Brigatinib, Imatinib, and Sorafenib. Sorafenib, a multitargeted kinase inhibitor, directly blocks tumor cell proliferation by disrupting the RAF/MEK/ERK signaling cascade. It also inhibits tumor angiogenesis indirectly by targeting VEGF and PDGF. Clinically, it is a targeted agent for the treatment of advanced liver and kidney cancer [185, 186]. However, drug resistance and tumor recurrence are still important factors that restrict the use of Tkis. It is still necessary to explore the mechanism of human resistance to Tkis and tumor recurrence and seek better treatment strategies.

### Targeting the PI3K-AKT-mTOR signaling pathway

The PI3K-AKT-mTOR (phosphocarnosine 3-kinase-AKT-mammalian target of rapamycin) pathway is a crucial signaling pathway that regulates essential processes including cell growth, differentiation, inflammation, and metabolism [187, 188]. Phosphocarnosine 3-kinase (PI3K), a protein family consisting of lipoenzymes, has a major substrate--AKT. When AKT is activated by PI3K, it exhibits kinase activity by mediating many cellular phenotypes such as survival and proliferation [188, 189]. A number of studies have revealed the potential dysregulation of PI3K-AKT-mTOR pathway in tumors. At the same time, gene mutations in this pathway can frequently enhance the proliferation and migration of tumor cells. Targeting mTOR with inhibitors such as rapamycin and other small molecules has shown promising therapeutic effects in many clinical trials [189–191]. In our previous discussion, phosphorylation at Ser199 of *HNRNP A1* was activated by AKT, and rapamycin was able to activate cyclin D1/c-myc IRES by targeting the AKT mTOR-*HNRNP A1* (phosphorylation) signaling axis. This indicates that the phosphorylation level of *HNRNP A1* protein and the activity of cyclin D1/c-myc IRES can determine the sensitivity and resistance of tumor cells to rapamycin [48]. PP242 (Torkinib), another inhibitor targeting mTOR, which can also promote the activity of cyclin D1/c-myc IRES. GSK3235025 is a potent inhibitor of PRMT5. The methylation of *HNRNP A1* protein Arg-218/225 mediated by PRMT5 is functionally antagonistic to Ser199 phosphorylation, and GSK3235025 can reduce the activity of cyclin D1/c-myc IRES. The combination of GSK3235025 and PP242 has a remarkable effect on GBM in vitro [49]. LI et al. showed that the positive feedback regulation formed by AKT and mTORC2 can be inhibited by Torin 1 (another mTOR-targeted inhibitor), thereby inhibiting the expression of AKT and ultimately inhibiting the phosphorylation of *AUF1* in liver cancer cells, while *AUF1* can promote the membrane localization and phosphorylation of AKT. Therefore, *AUF1*-targeting activators may promote cancers in which the mTORC2-AKT pathway is upregulated, and it is valuable to further investigate whether they can be combined with Torin-1 drugs to treat such cancers [77]. AKTi-1/2 is a quinoxoline compound. Ngoc T. Vu et al.'s study found that AKTi-1/2 can control the variable splicing of Caspase9 by inhibiting the phosphorylation of *HNRNP L* and promoting the up-regulation of the expression of Caspase9a subtype, thus promoting the apoptosis of lung cancer cells [119].

PI3K, AKT, and mTOR regulate each other to drive the development of cancer. Several inhibitors such as rapamycin, PP242, and AKTi-1/2 regulate the phosphorylation level of HNRNP family molecules by targeting the PI3K-AKT-mTOR axis, thereby inhibiting the proliferation, migration, and survival of cancer cells. In addition, many inhibitors targeting the PI3K-Akt-mtor axis have been developed, PI3K inhibitors that have been put into clinical use include AMG319, Alpelisib, Copanlisib, etc. Akt inhibitors include Afuresertib, Ipatasertib. The mTOR inhibitors include Temsirolimus, Everolimus, etc [192]. At present, the effect of single inhibitor is often weak, and it can easily lead to drug resistance. The combination of drugs to inhibit the PI3K-Akt-mTOR axis remains a hot research topic, but the potential side effects of combining these specific inhibitors still warrant careful consideration. Additionally, PI3K-AKT-mTOR axis not only plays a crucial role in cancer cells but also has important functions in normal cells. Achieving truly specific targeted therapy and effective drug combinations remains a challenge.

### Targeting cyclin kinase

Multiple complex processes work together to regulate the cell cycle, and these processes are controlled by multiple protein factors. CDKs are the core kinase family that regulates the normal progression of the cell cycle and their functions are regulated by cell cycle checkpoints. Targeting the regulation of cell cycle and checkpoint mechanism in tumor cells has become an important potential direction for anticancer therapy [193–195]. In glioblastoma, the intricate interaction between phosphorylation and methylation of *HNRNP A1* results in modified activity of cyclin D1 [48, 49]. The development of drugs that selectively suppress the activity of cyclin D1 may potentially block or control this altered activity, perhaps offering promising prospects for the treatment of glioblastoma. In NSCLC, activation of *HNRNP A0* by MK2 stabilizes Gadd45α and p27 mRNA, which promotes checkpoints and hinders the cell cycle progression [42].

We found that these cell cycle factors are regulated by post-translational modified HNRNPs molecules, and the design of inhibitors targeting these modified HNRNPs may be a feasible approach to targeted therapy.

### Targeting strategies for SUMOylation

SUMOylation has the function of stabilizing protein molecules and plays a key role in tumor EMT, metastasis, treatment resistance, and anti-tumor immune response. Meanwhile, the SUMOylation of HNRNP family members can enhance the stability of signaling pathways and effectively enhance cell activity [196]. Targeting SUMO E1 activating enzymes, E2 conjugating enzymes, or E3 ligases can block the SUMOylation cascade and suppress the stability of specific oncogenic molecules. TAK-981, a small molecule SUMO E1 inhibitor. Guo et al’s research has interpreted the potent anti-tumor ability of TAK-981, which can effectively inhibit the SUMOylation of *HNRNP A2/B1* and inhibit the malignant trend of GBM. However, its poor penetration of the blood-brain barrier in humans remains a limiting problem [56].

TAK-981 activates the IFN signaling pathway in immune cells and has been shown to promote the innate immune response and the activation of innate immune cells, including NK cells, macrophages, dendritic cells, and T cells [197]. TAK-981 is the first E1 SUMO activation enzyme inhibitor to enter clinical trials. Compared to traditional SUMOylation inhibitors like ML-792 and ML-93, TAK-981 can achieve higher efficacy at lower doses [198]. In the future, drug studies targeting SUMOylation will help us understand the mechanism of SUMOylation in cancer progression, which will provide innovative strategies for cancer treatment. In malignant tumors, inhibition of upstream molecules that activate SUMOylation may be an indirect direction to target carcinogenic HNRNPs-SUMO, but the specificity of drugs and the resistance of cancers are still limited problems.

### Hormonotherapy

A final treatment option that must be mentioned is hormonotherapy, which is often considered an adjuvant therapy for radiotherapy and surgery. Hormone therapy for prostate cancer is relatively well-established. In the previous discussion, cyproterone acetate (CPA) is a steroid anti-androgen. CPA mediates the hyperphosphorylation of *HNRNP K* in vivo and increases the tight binding of *HNRNP K* to androgen receptor (AR), which is often used in the clinical treatment of advanced prostate cancer [148]. In addition, several hormone therapy drugs are gradually used in the clinic, such as abiraterone, enzalutamide, and apalutamide. However, many common adverse reactions still limit the widespread use of these drugs.

## Conclusion and prospects

Developing cancer treatment strategies based on PTMs of HNRNPs molecules still remains significant challenge. Firstly, the diverse phenotypes of cancer cells caused by the crosstalk of various PTMs are complex. PTMs are increasingly recognized as only a small part of the protein network regulation. Precise targeting within the highly dynamic and complex post-translational modification process is a key focus of targeted therapy. Secondly, besides common modifications, further researches are needed to investigate the regulatory mechanisms of novel and unknown modifications. Moreover, in preclinical studies of various cancer treatments, there is a lack of efficacy and safety in targeting these PTMs. In conclusion, developing effective therapeutic strategies directed against HNRNPs-based PTMs requires a comprehensive understanding of the complex regulatory networks involved in multiple cancers, while minimizing damage to normal human cells.

**Fig. 3:**
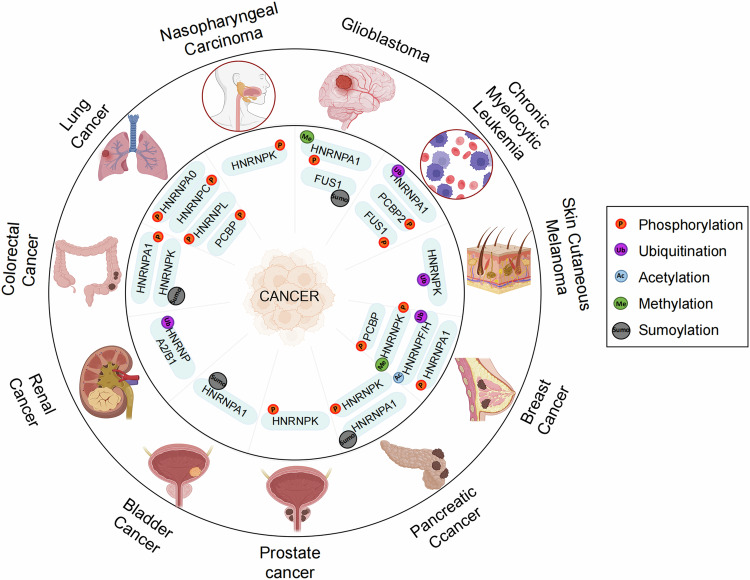
Overall summary of the regulation of HNRNPs by PTMs in cancer (created with BioRender.com). As shown in this figure, the outer circle represents each cancer, and the inner circle is the PTMs type possessed by the HNRNPs corresponding to each cancer. On the right, we add an explanatory text to explain the abbreviations (P, Ub, Ac, Me, Sumo).

In this context, we have elucidated not only the different modifications of HNRNPs in cancers (Fig. 3) but also the different mechanisms caused by them. Additionally, we anticipate that the discovery of new post-translational modifications (PTMs) and regulatory pathways will provide new insights into cancer development, thereby offering innovative approaches for targeted cancer therapy.
